# Vocal assessment in patients submited to CO_2_ laser cordectomy

**DOI:** 10.1016/S1808-8694(15)30960-5

**Published:** 2015-10-19

**Authors:** Leonardo Haddad, Márcio Abrahão, Onivaldo Cervantes, Fábio Pupo Ceccon, Ingrid Gielow, Jomar Rezende Carvalho, Fernando Danelon Leonhardt

**Affiliations:** a ENT specialist, post-graduate student at the Otorhinolaryngology and Head & Neck Surgery Unit at UNIFESP; b Lecturer of the Otorhinolaryngology and Head & Neck Surgery Unit at UNIFESP; c Lecturer of the Otorhinolaryngology and Head & Neck Surgery Unit at UNIFESP; d Doctor in Otorhinolaryngology and Head & Neck Surgery at UNIFESP, Collaborating medical doctor; e Doctor in Human Communication Disorders at the Sao Paulo Federal University - UNIFESP-EPM. Phonoaudiologist of the Head & Neck Surgery Department - UNIFESP; f ENT specialist, post-graduate student at the Otorhinolaryngology and Head & Neck Surgery Unit at UNIFESP; g ENT specialist, post-graduate student at the Otorhinolaryngology and Head & Neck Surgery Unit at UNIFESP. This study was conducted in the Head & Neck Surgery Unit at Sao Paulo Federal University - UNIFESP-EPM, as a Master's degree

**Keywords:** laringectomy, lasers, voice quality, voice

## Abstract

**Aim:**

To evaluate voice outcomes in patients with early glottic carcinoma treated by CO_2_ laser cordectomy.

**Method:**

15 patients with glottic Tis and T1 squamous cell carcinoma treated with CO_2_ laser were analyzed. The assessment consisted of perceptual voice analysis, objective voice evaluation and video-laryngo-stroboscopic exam. In addition, patients rated their voices and completed the Voice related Quality of Life (VR-QOL) questionnaire. The results were compared with those obtained in a matched control group.

**Results:**

Most of the patients presented some degree of hoarseness on perceptual voice analysis, mainly rough and breathy voices. Their acoustic evaluation compared with the control group showed a small increase in fundamental frequency, but with no statistically significant difference, and the values of jitter, shimmer and noise to harmonic ratio were worse and statistically significant. As regards to video-laryngo-stroboscopic findings, better results were achieved in the less extensive resection group. Patients have had minimal repercussion in their life quality in respect to voice. Conclusions: In spite of voice alterations in patients submitted to cordectomy by CO_2_ laser, functional results are acceptable, with minimal repercussion in their quality of life. Avaliação da voz em pacientes submetidos à cordectomia com laser de CO_2_.

## INTRODUCTION

Cancer of the larynx is the second most frequent tumor of the upper airway and upper digestive tract, second only to oral cavity tumors. Brazil occupies the second place in the world in the incidence of cancer of the larynx. This disease affects mostly males in the sixth and seventh decades of life^1^. Most of the malignant tumors of the larynx (95%) are squamous cell carcinomas (SCCs) which belong to the epithelial lineage^2^.

Initial glottic carcinomas, classified as in situ carcinomas (Tis) and T1, may be treated by open surgery (cervical access), radiotherapy and laryngeal microsurgery with conventional instruments or by CO_2_ laser. These approaches provide satisfactory local control and similar survival rates^3^, ^4^, ^5^.

Cordectomy is the surgical procedure used for early glottic tumors, where the affected vocal fold is resected at varying depths.

Since Strong & Jako introduced CO_2_ laser in laryngeal surgery in 1972^7^, this therapeutic strategy has gained worldwide acceptance for the treatment of laryngeal tumors, particularly for early glottic tumors^3^, ^4^, ^8^, ^9^, ^10^, ^11^, ^12^, ^13^, ^14^, ^15^.

Cure rates for CO_2_ laser treated patients with early glottic tumors vary from 89% to 100%^4^, ^14^, ^16^, ^17^, ^18^ and are similar to radiotherapy cure rates^19^, ^20^.

The advantages of CO_2_ laser compared to other treatment strategies include lower morbidity compared to radiotherapy side effects (mucositis, xerostomia and dental problems), lower cost as patients do not require traqueostomy, improved homeostasis, and the fact that CO_2_ laser does not exclude other forms of treatment if the disease recurs locally, regionally or at a distance^5^, ^19^.

When different treatment strategies offer similar cure rates, resulting voice quality becomes paramount in the therapeutic decision. There are few published papers in medical literature about voice following laser cordectomy for the treatment of laryngeal tumors.

Most of the studies compare cordectomy with radiotherapy, and results are controversial. Some authors have reported worse outcomes in patients undergoing laser surgery, which may limit its use^21^, ^22^, ^23^. Other studies, however, have stated that voice quality in patients undergoing CO_2_ laser cordectomy is similar to that in radiotherapy patients^24^, ^25^, ^26^, ^27^.

CO_2_ laser cordectomy provides improved results in the voice quality of patients, compared to conventional cordectomy^28^, ^29^, ^30^. Few studies specifically on voice assessment in patients that have undergone CO_2_ laser cordectomy have been published to date.

The factors that stimulated this study where the importance of preserving the phonatory function when treating early glottic tumors and the lack of guidelines on voice quality in patients undergoing CO_2_ laser cordectomy for the treatment of laryngeal tumors.

Therefore, the aim of this study was to assess voice quality in patients with stages Tis and T1 glottic carcinoma undergoing CO_2_ laser cordectomy.

## METHODS

Between January and May 2004 we assessed fifteen patients (12 males and 3 females) who had undergone CO_2_ laser cordectomy for the treatment of early vocal fold SCC. Early glottic SCC was defined in this study as patients with vocal fold Tis and T1 tumors. Patients were referred to us from the Head and Neck Surgery Unit at Sao Paulo University - Paulista School of Medicine.

Ages varied from 43 to 82 years, average 63.1 years. Average follow-up time was 14.6 months, varying from 3 to 38 months.

Patients were staged, based on data in their charts, using the clinical classification of the International Union Against Cancer (UICC) and the American Joint Committee on Cancer (AJCC) 2002.

Three patients were staged as TisN0 and twelve as T1aN0. Types of cordectomy were classified into five types according to the European Laryngological Society^6^ guidelines, namely type I - sub-epithelial resection, type II - subligamentar resection, type III - transmuscular resection, type IV - total cordectomy, and type V - extended cordectomy. Type V is subdivided into Va (resection of part of the contra-lateral vocal fold), Vb (resection of the arytenoid), Vc (resection of the ventricular band), and Vd (resection of part of the subglottis). Three patients underwent type I cordectomy (Tis), five patients underwent type II cordectomy, four patients underwent type III cordectomy, and three patients underwent type IV cordectomy. No patient required type V cordectomy.

Surgery was done under general anesthesia. A 400mm lens microscope was coupled to a Visograf® CO_2_ laser unit. A 5 to 7 Watts superpulse mode 0.5 mm spot was used. Safety margins were 1 to 2mm. Following tumor resection upper, lower, anterior, posterior and deep margins were sent to frozen section examination, to assure surgical safety margins.

All patients included in this study had been operated at least three months before enrollment, and were referred to post-operative speech therapy. No patient showed signs of local and/or regional recurrence and none had a second primary tumor.

Patients underwent subjective and objective voice assessment and videolaryngostroboscopic evaluation between the 3rd and 38th post-operative month. Subjective testing was done by perceptive-auditory voice analysis according to the GRBASI scale^32^, Sittel et al's (1998)^33^ general voice assessment numerical scale and Behlau's^35^, ^36^ Portuguese adaptation of Hogikyan & Sethuraman's (1999) instrument for voice-related quality of life (V-RQOL).

GRBASI's scale assesses the global impression of dysphonia (G) considering hoarseness (R), soprosity (B), asteny (A), tension (S) and instability (I), which are scored 0 to 3, 0 (no change), 1 (mildly altered), 2 (moderately altered) and 3 (severely altered). V-RQOL is a protocol whereby patients relate their voice to quality of life in a 0 to 100 score where 100 is improved quality of life^35^, ^36^. In a general voice assessment, patients classify their voice in a 0 to 5 scale where 0 is impaired voice and 5 is normal or near normal voice.

Computer analysis was made based on open and isolated phonation of the sustained vowel /e/ picked up by an AKG model C410 professional microphone at a standard 5 cm distance from the patient. Voice recording was done by the Computerized Speech Lab - CSL, model 4300B, and analyzed by the Kay Elemetrics Corp.'s MDVP (Multi-Dimensional Voice Program) voice analysis program.

Each patient was asked to emit an isolated and sustained vowel /e/. A voice sample was considered as the most stable 3 second period, whenever possible eliminating the beginning and the end of sound emission or a maximum period of voice stability. The following acoustic measurement parameters were used in this study: fundamental frequency, the perturbation quotients Jitter (variations of frequency) and Shimmer (variations of amplitude), and noise measurements (harmonics-to-noise ratio).

Computer analysis compared fundamental frequency, jitter, shimmer, and harmonics-to-noise ratio with a patient control group (average age 57.6 years) that had normal larynxes, and no complaints about voice.

Videolaryngostroboscopy was used to analyze the following parameters:

1. Glottic coaptation: complete or incomplete
2. Laryngeal vestibule constriction: normal or with hyperconstriction.
3. Presence of vibration: absent, glottic, supraglottic or combined. Vibration was considered as absent if there was any area of akinesia in the resected glottic region, with no supraglottic compensation.

The Stata 7.0 program was used for statistical analysis. Data had a normal distribution allowing us to use Student's t-test for non-related samples to compare numerical data in the study and the control groups. The significance level was 0.10.

## RESULTS

Student's t-test was used to compare the average fundamental frequency of males in the study group and the control group (139.3Hz). The descriptive value was 0.2008, which suggests no differences between both groups. There were not sufficient females in the study group to apply statistical tests. Descriptively, however, the 226.24 Hz average value may be compared to the average control group value (202,23 Hz).

Student's t-test for independent samples demonstrated that average jitter in patients was higher than in the control group (p = 0.001), that average shimmer in patients was higher than in the control group (p = 0.0007), and that patients had a higher average harmonics-to-noise ratio compared to the control group (p = 0.053).

## DISCUSSION

There is no agreement about voice quality after different treatment strategies for glottic tumors, particularly Tis and T1 tumors. Important factors influencing the decision for a specific treatment procedure, except for voice quality, include oncological results, cost and morbidity.

There are few papers in literature assessing voice following CO_2_ laser cordectomy, and only rarely is there a comparison with a control group of normal individuals; there are no such papers published in Brazil to date. The importance of voice is evident as we measure voice changes in patients undergoing CO_2_ laser cordectomy compared to normal individuals and to how much this affects their lives. Furthermore, future comparisons may be made both within groups undergoing similar treatment and between groups subjected to other strategies such as radiotherapy and conventional surgery.

In our patients age ranged from 43 to 82 years (average 63.1 years), with 80% males. This is similar to other published studies, evidence that malignant laryngeal tumors are more frequent in men in the sixth and seventh decades of life^1^.

Although all patients were staged as Tis and T1a, the type of cordectomy varied in the group. Less extensive cordectomies, classified as type I and II, were done in 53% of patients. Type III and IV cordectomies were done in the remaining 47%.

Subjective analysis demonstrated that the majority of patients had some degree of dysphonia, usually hoarseness and soprosity. These results are not surprising, as hoarseness (R) is related to vocal crepitation and bitonality due to irregular vibration of the vocal folds, and soprosity (B) is related to air escape during phonation. One naturally expects a scar and local irregularity when cordectomy is done, as part of the vocal fold is removed.

**Table 1:** Results of the GRBASI scale.

Variable	0	1	2	3	total
G	3	3	4	5	15
R	5	3	4	3	15
B	3	3	6	3	15
A	12	2	1	0	15
S	12	1	0	2	15
I	6	4	4	1	15

**Table 2:** Patient distribution considering the general voice assessment and the type of cordectomy (0 is impaired voice and 5 is normal or near normal voice).

	General assessment					
	Patient	1	2	3	4	5
Type I or II	1					√
	2					
	3				√	
	4					√
	5					√
	6				√	
	7					√
	8		√			

Type III or IV	9				√	
	10					√
	11					√
	12			√		
	13			√		
	14		√			
	15			√

**Table 3:** V-RQOL (voice and quality of life) protocol scores, related to the type of cordectomy.

Patient	Type	V-RQOL
1	I	90,0
2	I	87,5
3	I	100,0
4	II	97,5
5	II	100,0
6	II	87,5
7	II	87,5
8	II	65,0
9	III	100,0
10	III	95,0
11	III	92,5
12	III	67,5
13	IV	90
14	IV	85
15	IV	77,5
	Average	88,17

**Table 4:** Results of acoustic voice analysis in patients undergoing CO_2_ laser cordectomy, considering the type of surgery and a comparison with the control group.

Patient	Type	Fund. freq. (Hz) male	Fund. freq (Hz) female	Jitter%	Shimmer%	Ratio harm/noise
1	I		191,72	1,59	3,34	0,13
2	I	120,27		0,99	4,55	0,14
3	I		205,25	1,61	1,97	0,10
4	II	172,68		3,09	3,12	0,09
5	II	143,47		1,49	2,36	0,13
6	II	140,06		2,15	4,43	0,16
7	II	161,89		2,62	3,09	0,13
8	II	124,85		8,04	12,72	0,47
9	III		281,75	2,82	3,26	0,09
10	III	119,77		3,25	5,05	0,13
11	III	158,04		1,24	5,48	0,13
12	III	174,40		4,24	11,74	0,29
13	IV	226,67		5,89	15,99	0,44
14	IV	112,00		20,85	24,59	0,78
15	IV	356,00		5,97	15,24	0,60
	Average	167,51	226,24	3,18	7,79	0,25
	Average Control	139,30(p=0,2008)	202,23	0,76(p=0,001)	3,95(p=0,0007)	0,13(p=0,053)

**Table 5:** Videolaryngostroboscopic images, considering the type of cordectomy.

Patient	Type	Coaptation	Constriction of vestibule	Presence of vibration
1	I	Complete	Normal	Glottic
2	I	Complete	Normal	Glottic
3	I	Incomplete	Normal	Glottic
4	II	Complete	Normal	Combined
5	II	Complete	Normal	Glottic
6	II	Incomplete	Normal	Combined
7	II	Complete	Normal	Combined
8	II	Complete	Hyperconstriction	Supraglottic
9	III	Incomplete	Normal	Absent
10	III	Complete	Normal	Glottic
11	III	Incomplete	Hyperconstriction	Glottic
12	III	Complete	Hyperconstriction	Absent
13	IV	Incomplete	Hyperconstriction	Absent
14	IV	Complete	Hyperconstriction	Absent
15	IV	Incomplete	Hyperconstriction	Supraglottic

Patient opinions about their voice following treatment were also of interest in this study. Using Sittel et al's (1998)^33^ scale, six patients (40%) who judged their voices as normal or near normal, four patients (26.7%) judged their voices as good for communicating but pathological, three patients (20%) judged their voices as reasonable, and only two (13.3%) judged their voices as impaired, but understandable. No patient classified their voice as very dysfunctional.

The impact of voice on the patient's quality of life should be a constant concern in the treatment of laryngeal tumors. This concept, however, is recent; Zeitels (1995)^12^ states that the current standard of phonomicrosurgery is to obtain oncological radicality with voice quality that causes the least change in quality of life. Classical assessments of health treatment valued only the presence or absence of disease following treatment, which is important, but insufficient to assess the impact on life in general^37^. O V-RQOL was developed specifically to correlate how much voice change may affect the patient's quality of life^36^. In this study the average V-RQOL was 88.17. These results show that although most patients undergoing CO_2_ laser cordectomy had some degree of dysphonia, the effect on activities of daily life was minor. It is important to underline that no patient in this study used his or her voice professionally, which reduces the impact of voice changes on the quality of life. In fact, the V-RQOL is not a sensitive method to assess professional voice users^36^, as minor changes in a singer, for instance, may compromise his or her career, whereas the same change in a person that does not use his or her voice professionally may cause minimal impact.

Stoeckli et al (2001)^38^ used the EORTC QLQ-C30 quality of life questionnaire and the head & neck module (EORTC QLQ-H&N35) developed by the European Organisation for Research and Treatment of Cancer for patients with early laryngeal tumors treated with radiotherapy and CO_2_ laser surgery. Results confirm good quality of life in both treatment strategies. Specific head & neck questionnaire questions about swallowing solid food, xerostomy and dental problems scored worse for patients undergoing radiotherapy, although there was no difference in voice quality. Schneider, Guidicelli & Stöckli in 2000^39^ have also described similar results.

Computer voice acoustic analysis using the Kay Elemetrics Corp. Computerized Speech Lab-CSL model 4300 MDVP (Multi-Dimensional Voice Program) voice analysis program showed that, except for the fundamental frequency, all voice acoustic analysis parameters in this study - percentage of jitter and shimmer, and harmonics-to-noise ratio - are significantly affected by CO_2_ laser cordectomy (p< 0.1) comparing results of the surgery group to controls.

The average fundamental frequency for operated male patients was higher compared to controls, although this difference was not statistically significant. There were not sufficient females in the study group to apply statistical tests, but a minor increase of 24.01 Hz in the fundamental frequency was seen compared to controls (202.23Hz). Similar results have also been reported by McGuirt et al. (1992)^31^ with a slightly higher average fundamental frequency compared to a normal control group. Voice tends to become more acute due to the removal of tissue and resulting compensation mechanisms. The main factors affecting the fundamental frequency are the length, mass and tension of the vocal fold; the lower the mass, the higher the frequency. Furthermore these patients frequently compensate with hyperconstriction and tension which can also increase the fundamental frequency.

Both jitter and shimmer were significantly higher in the study group compared to controls. These results reflect increased aperiodicity in the glottic cycle and sound wave variability. The harmonics-to-noise ratio is directly related to voice quality^36^. It analyzes aperiodic components of sound and is considered a good correlate to what we consider as dysphonia. The study group average was higher compared to controls and was statistically significant (p = 0.053).

Results of objective voice analysis clearly suggest that voice quality in patients undergoing CO_2_ laser cordectomy is compromised compared to individuals with no voice-related problems. A further issue is that when these patients seek medical help, they already have some degree of dysphonia by having a glottic tumor. Pre-operative voice assessment was not evaluated in this study, but is suggested for future studies.

Theoretically, regardless of the treatment, voice quality of patients with glottic tumors will never be equal to their voice before the disease. Even the smallest cordectomy will involve resection of part of the mucosa, reducing the ability for vibration at that point.

Although considered a more conservative strategy, radiotherapy also has negative effects on voice quality compared to controls^40^. Hocevar-Boltezar et al. (2000)^41^ and Dagli et al. (1997)^42^ also found increased jitter and shimmer values and a higher fundamental frequency compared to controls in irradiated patients assessed objectively. Such results may be explained by post-radiation vocal fold fibrosis and rigidity in both sides.

Videolaryngostroboscopy found that 75% (n=6) of patients undergoing type I and II cordectomy presented complete glottic closure. In this same group glottic mucosal vibration was always present; only 1 patient (individual number 8) had supraglottic vibration. This same patient was the only one in this group (cordectomy types I and II) that had laryngeal vestibule hyperconstriction. All subjective and objective parameters in this patient, except for the fundamental frequency, were worse compared to other patients in the same group. A further interesting point is that even in type II cordectomy, glottic closure was complete in 4 of the 5 operated patients. This is relevant because voice quality is intimately related to glottic closure. Frequently in situ carcinoma is treated with type I cordectomy, and the surgeon may find himself surprised with the pathological examination showing compromised surgical resection margins. The next step would be to widen the surgical margins, which means the patient has to undergo a second operation or be referred to radiotherapy. Knowing that the post-operative functional result is similar in type I and II cordectomies, a safer and wider margin procedure for Tis could be advised.

Videolaryngostroboscopy of type III and IV cordectomies showed that 57.1% (n=4) of patients presented incomplete glottic closure, an increased trend to have laryngeal hyperconstriction as a compensation mechanism and no mucosal vibration. Stroboscopic changes correlated with voice quality data in study group patients, probably due to the amount of tissue removed, decreased mucosal vibration and compensation mechanisms.

Although results suggest that patients undergoing CO_2_ laser cordectomy have abnormal voice parameters, the possibility of preserving communication is itself reason for satisfaction. Frequently patients lose motivation when receiving a diagnosis of cancer of the larynx, believing that treatment is necessarily related to voice loss, which is so important for social interactions. Expectations of voice professionals may not be reached, but this is not what happens to most patients; they frequently surprise us by being satisfied with their resulting voice quality.

## CONCLUSION

Voice perceptive-auditory analysis indicates that most patients undergoing CO_2_ laser cordectomy presented some degree of dysphonia, usually hoarseness and soprosity.

Both the general voice assessment made by patients and the quality of life protocol scores suggest that CO_2_ laser cordectomy produces acceptable functional results, with minor repercussion on the patient's lives.

Acoustic voice analysis showed a minor increase in the fundamental frequency of operated patients compared to controls. This difference was not statistically significant. Worse jitter, shimmer and the harmonics-to-noise ratio results were seen in patients undergoing CO_2_ laser cordectomy.
